# Engineering of an *angiogenic niche* by perfusion culture of adipose-derived stromal vascular fraction cells

**DOI:** 10.1038/s41598-017-13882-3

**Published:** 2017-10-27

**Authors:** Giulia Cerino, Emanuele Gaudiello, Manuele Giuseppe Muraro, Friedrich Eckstein, Ivan Martin, Arnaud Scherberich, Anna Marsano

**Affiliations:** Departments of Biomedicine and Surgery, University of Basel and University Hospital of Basel, 4031 Basel, Switzerland

## Abstract

*In vitro* recapitulation of an organotypic stromal environment, enabling efficient angiogenesis, is crucial to investigate and possibly improve vascularization in regenerative medicine. Our study aims at engineering the complexity of a vascular milieu including multiple cell-types, a stromal extracellular matrix (ECM), and molecular signals. For this purpose, the human adipose stromal vascular fraction (SVF), composed of a heterogeneous mix of pericytes, endothelial/stromal progenitor cells, was cultured under direct perfusion flow on three-dimensional (3D) collagen scaffolds. Perfusion culture of SVF-cells reproducibly promoted *in vitro* the early formation of a capillary-like network, embedded within an ECM backbone, and the release of numerous pro-angiogenic factors. Compared to static cultures, perfusion-based engineered constructs were more rapidly vascularized and supported a superior survival of delivered cells upon *in vivo* ectopic implantation. This was likely mediated by pericytes, whose number was significantly higher (4.5-fold) under perfusion and whose targeted depletion resulted in lower efficiency of vascularization, with an increased host foreign body reaction. 3D-perfusion culture of SVF-cells leads to the engineering of a specialized milieu, here defined as an *angiogenic niche*. This system could serve as a model to investigate multi-cellular interactions in angiogenesis, and as a module supporting increased grafted cell survival in regenerative medicine.

## Introduction

The rapid development of a reproducible vascular network in (three-dimensional) 3D engineered tissues is paramount for guaranteeing their viability and proper function upon implantation^1,2^.

Common *in vitro* strategies aim to promote the vascularization of engineered tissues by 1) using growth factors–releasing scaffolds^3,4^, 2) co-culturing mature endothelial cells (EC)^5,6^, or bone marrow-/adipose tissue stromal cell-derived endothelial progenitors cells (EPC) with mesenchymal stem/stromal cells (MSC) or perivascular cells^7,8^, or 3) using pre-formed micro-fabricated engineered vasculature^9^. Despite being valid approaches, these strategies present some weaknesses. Indeed, pitfalls in i) matching growth factor type and time-releasing profile^10^, ii) identifying the proper cell types and their ratio^11^, and iii) selecting suitable fluid shear stresses (SS) within the micro-scaffold^12^ are still unsettled. Moreover, an *in vitro* 3D model able to summarize the key components of the angiogenic process, like the dynamic interplay between EC and other vascular/mural cells (e.g. smooth muscle cells, pericytes and MSC)^13,14^, the supporting extracellular matrix (ECM) and/or the basement membrane deposition, and the exposure to the blood hydrodynamic-based shears^15,16^, does not yet exist^11,17^.

Concerning the cell choice, the adipose tissue-derived stromal vascular fraction (SVF) is originally composed by multiple cell types. Indeed, the SVF heterogeneity, mainly constituted by EC, perivascular cells and MSC^18,19^, confers to this cell collection, among many others, a prevailing vascular potential. Actually SVF cells, either when dynamically^20^ or statically cultured^21^, have demonstrated to be able of generating vascular-like networks in *in vitro* engineered tissues (e.g. bone, skin, and heart)^20,22,23^, and to promote the direct connection to the host vessels by anastomosing and/or the formation of new functional vessels by releasing angiogenic factors upon implantation^24–26^. Regarding the other cell subpopulations, especially pericytes have been shown to fulfill several important functions during the development and maintenance of preformed microvascular networks^18,27^.

Together with the cell source, the establishment of appropriate biochemical and physical cues during *in vitro* culture is also essential for engineering vascularized and viable clinically relevant tissue substitutes^28^. On one hand, the release of pro-angiogenic factors is recognized to enhance angiogenesis by inducing EC proliferation, matrix proteolytic activity, invasion into 3D matrices and formation of tubular structures^29,30^. On the other hand, the physical signals downstream of hemodynamic forces that regulate new blood vessel growth are equally relevant but still less understood^31,32^. *In vitro* models of vascular morphogenesis demonstrated that pre-exposure to wall SS enhanced the development of endothelial cord-like networks in a 2D matrigel-^33^ and 3D collagen- based^34^ models, proving the essential role of the flow for organizing EC into vascular structures.

In this study, we aim at developing a 3D multi-cellular engineered tissue (patch) able to *in vitro* recapitulate a complete and functional angiogenic microenvironment with a high vascularization potential *in vivo*. We hypothesize that the direct perfusion of the culture medium through the pores of 3D constructs will foster the growth of the EPC and the perivascular cell subpopulations and their organization in pre-capillary structures. The proactive angiogenic engineered microenvironment is then expected to promote the *in vivo* rapid vascularization of 3-mm-thick constructs, by integrating the main vascular building blocks: multi cell types, EC organization in capillary-like structures, newly deposited ECM backbone, molecular signals and physical cues.

## Results

**Figure 1:**
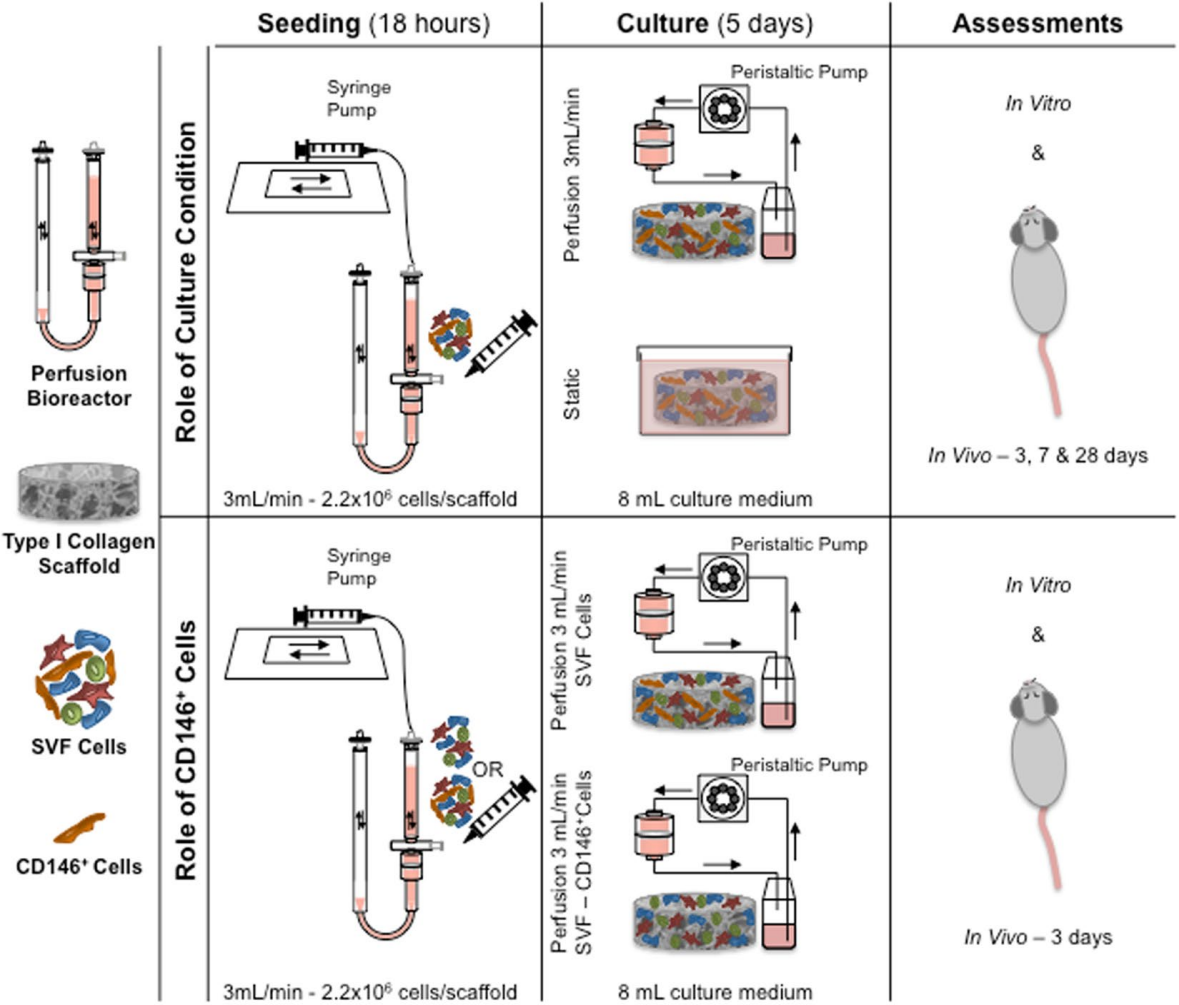
Scheme of the study. Summary of the main steps of the experimental plan.

In this study, we compared the effects of the direct perfusion and static culture on the heterogeneous SVF cell composition in terms of engineering a pro-angiogenic 3D environment (e.g. by increasing the endothelial/mural cell compartment, the release of angiogenic factors), and improving the *in vivo* angiogenic potential (Fig. 1). Perfusion culture was identified to significantly accelerate the vascularization of the SVF-based constructs, by means of the increased pericyte subpopulation (CD146^+^ cells). Thereafter, we investigated the role of pericytes in boosting the *in vivo* early angiogenesis and in modulating the host response by culturing in perfusion the whole SVF depleted of the CD146^+^ cells (Fig. 1).

### *In vitro* results

#### Perfusion increased ECM deposition, *in vitro* pre-vascularization and pro-angiogenic factor release

**Figure 2:**
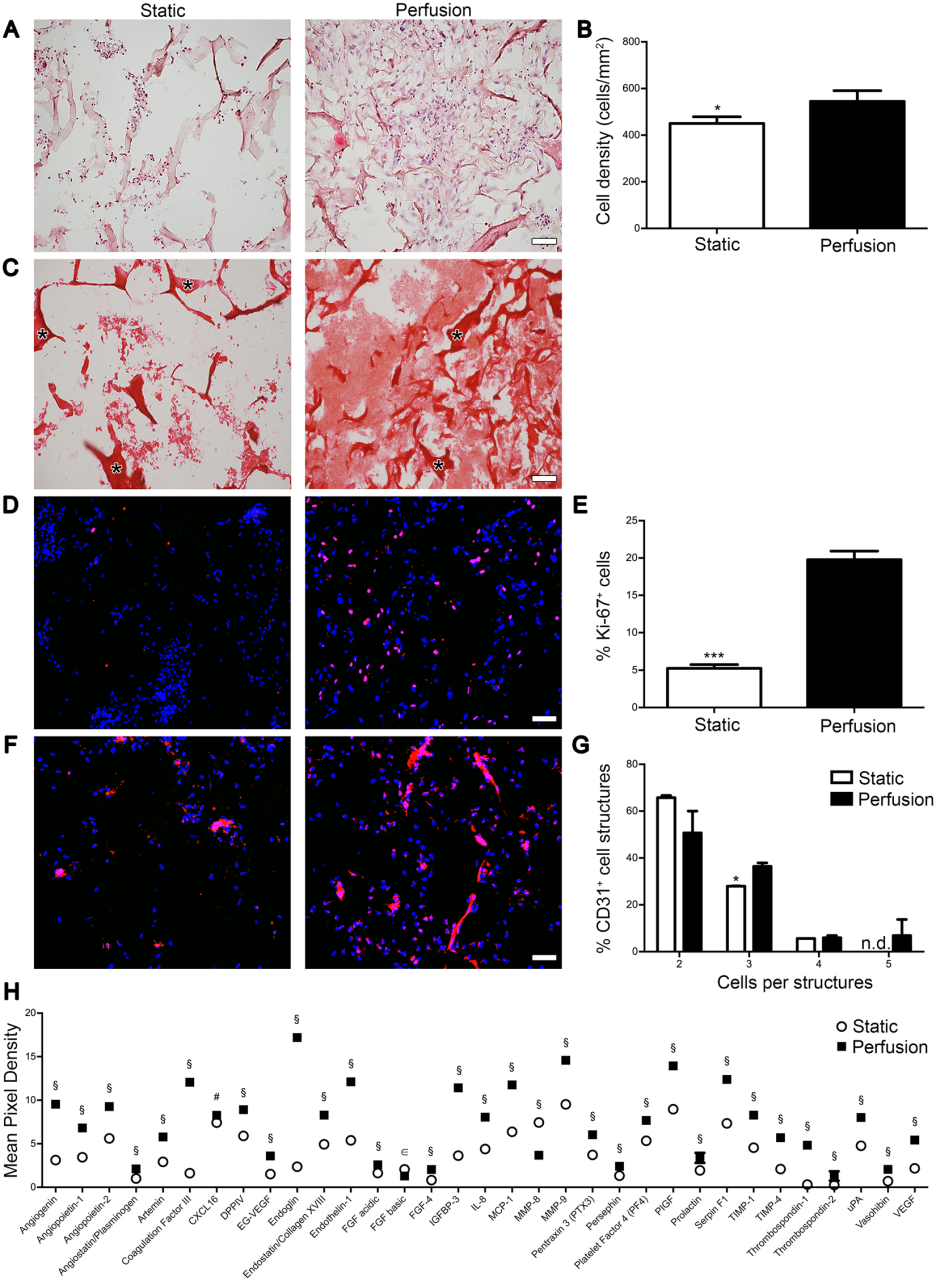
Perfusion increased ECM deposition, *in vitro* pre-vascularization and pro-angiogenic factor release. Analyses performed on constructs cultured 5 days either in perfusion or static condition. (**A**) Representative H&E images. (**B**) Cell density quantification. (**C**) Representative Picro-Sirius Red images. *Identifies collagen scaffold. (**D**) Representative immunofluorescence images for cell proliferative marker Ki-67 (red). Nuclei were stained with DAPI (blue). (**E**) Quantification of the proliferating cells stained with Ki-67 normalized over the total amount of DAPI. (**F**) Representative immunofluorescence images for endothelial marker CD31 (red). Nuclei were stained with DAPI (blue). (**G**) Percentage of endothelial cell structures. *p < 0.05, ***p < 0.001. n.d. = not detectable. (n donor = 3). (**H**) Human angiogenesis proteome profiler array showing the release of angiogenic factors. Data are presented as mean pixel density normalized on DNA. § = *** p < 0.001; # = ** p < 0.01; ϵ = * p < 0.05. Scale bar: 50 μm. (n donor = 2).

Following static culture, cells formed mainly aggregates not uniformly distributed throughout the construct. Scarce ECM was deposited among the cells leaving the scaffold pores mainly empty (Fig. 2A,C). Contrarily, direct perfusion fostered uniform cell distribution and abundant ECM deposition (Fig. 2A,C). The ECM was mainly composed of types I and III collagen as shown by the Picrosirius staining (Fig. 2C). The cell density was significantly higher in perfusion compared to static constructs (544.9 ± 46.3 and 450.6 ± 28.1 cells/mm^2^, respectively; Fig. 2B). Proliferating Ki-67^+^ cells were distributed uniformly throughout the perfused constructs (Fig. 2D) and significantly higher in percentage compared to static condition (19.7 ± 1.1 and 5.2 ± 0.5%, respectively; Fig. 2E). In static constructs, the majority of the EC formed small aggregates with few elongated cells organized in cord-like structures, whereas following perfusion culture EC established numerous cord-like structures (Fig. 2F). Complex vascular structures, formed by three or more aligned and elongated CD31^+^ cells, were present predominantly in the perfused constructs (Fig. 2G). Static and perfusion-conditioned culture media were collected and analyzed for secreted angiogenic factors (Fig. 2H, Supplementary Fig. S1A,S1B and Supplementary Table S1). Culture in perfusion up-regulated the 90% of the analyzed factors (Fig. 2H and Supplementary Fig. S1B), the majority of which is associated with a pro-angiogenic effect (51%: 16 out of 31), whereas 38% (12 out of 31) with an anti-angiogenic and 6% (2 out of 31) with an immunomodulatory effect. Human vascular endothelial growth factor (VEGF) was one of the most remarkably released under dynamic condition and enzyme-linked immunosorbent assay (ELISA) confirmed a release 3.25- fold higher than static culture (1281.8 ± 283.2 and 301.2 ± 29.8 pg/μg, respectively), which increased the expression of only 5.4% angiogenic factors (3 out of 55) (Fig. 2H and Supplementary Fig. S1B).

#### Perfusion enriched the SVF for vascular cell components

**Figure 3:**
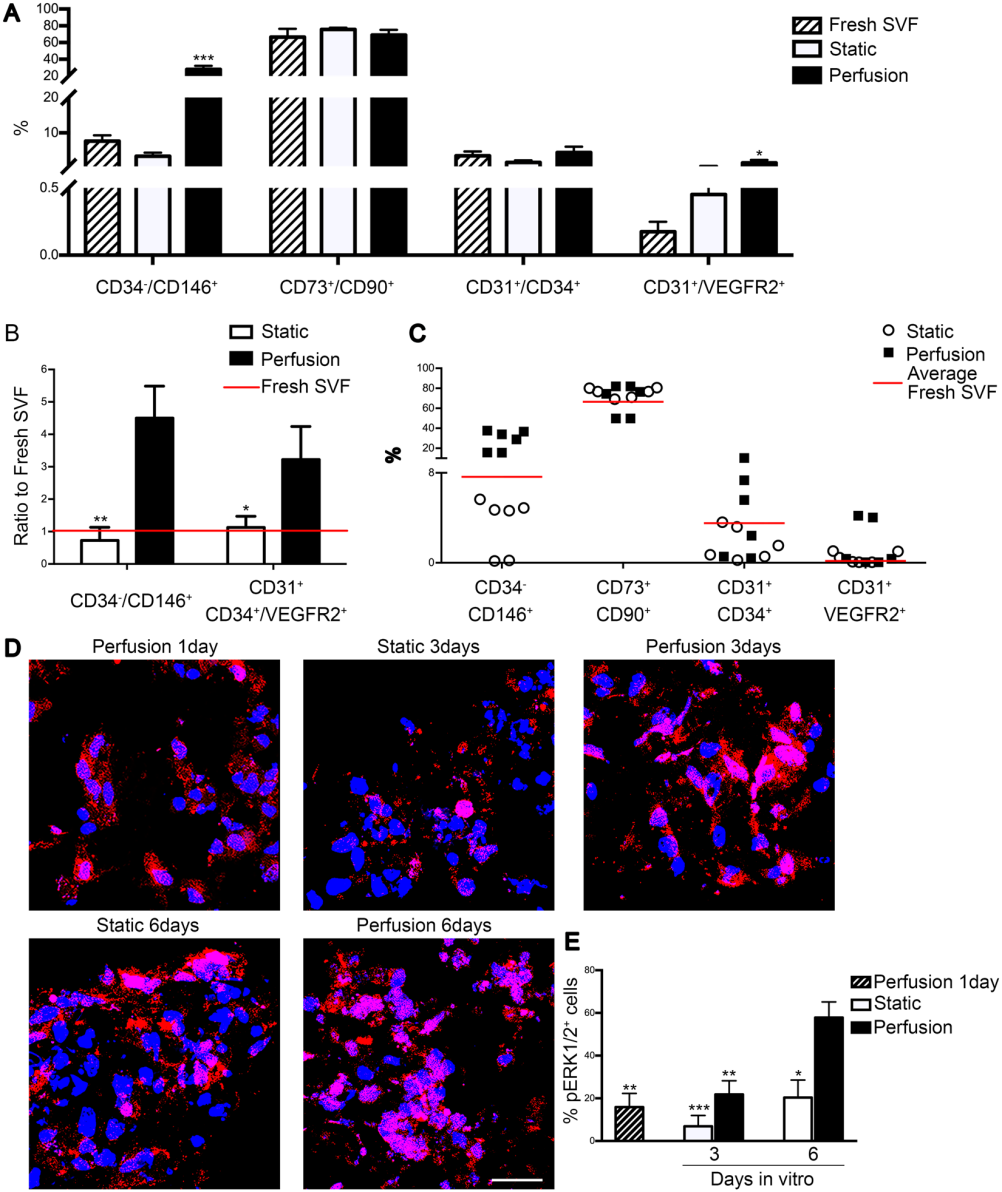
Perfusion enriched the SVF for the vascular cell component. All population presented are considered CD45^−^ (**A**–**C**). (**A**) *In vitro* cell phenotype characterization by 6-channel-flow-cytofluorimetric analysis of freshly isolated (Fresh) or SVF cells following static or perfusion culture. Quantification presented as percentage over the living cells (DAPI^+^) of different SVF subpopulations: pericyte (CD34^−^ CD146^+^), MSC (CD73^+^ CD90^+^), EC (CD31^+^ CD34^+^) and mature EC (CD31^+^ VEGFR2^+^) (n donor = 6). (**B**) Graph representing the ratio of pericytes and EC - progenitor and mature - over the fresh SVF (red line indicates ratio = 1). (**C**) Scatter plot showing the distribution of the 6 donors after static or perfusion culture (red lines indicate the mean). *p < 0.05, **p < 0.01, ***p < 0.001. (**D**) Representative immunofluorescence images for pERK(1/2) (red). Nuclei were stained with DAPI (blue). Scale bar = 20 μm. (**E**) Quantification of the pERK(1/2)^+^ cells normalized over the total amount of DAPI. *p < 0.05, **p < 0.01, ***p < 0.001 (n donor = 2).

The initial heterogeneity of fresh SVF cells was confirmed by the presence of MSC (66.6 ± 9.7% CD45^−^ CD73^+^ CD90^+^), pericytes (7.6 ± 1.6% CD45^−^ CD34^−^ CD146^+^), EPC (3.5 ± 1.2% CD45^−^ CD31^+^ CD34^+^), and mature EC (0.2 ± 0.07% CD45^−^ CD31^+^ VEGFR2^+^) (Fig. 3A). Static culture did not considerably modulate the percentage of the different subpopulations, whereas perfusion led to a significantly higher percentage of pericytes (28.1 ± 4.1%) and mature EC (1.5 ± 0.8%) compared to the freshly isolated SVF (Fig. 3A). Representative immunofluorescence for Ki-67 and CD31 confirmed indeed the proliferation activity of the endothelial cells (Supplementary Fig. S1D). Representative contour plots show how the pericyte subpopulation was clearly identified following 5-day perfusion culture in the CD45^−^, CD34^−^ and CD146^+^ quarter (Supplementary Fig. S1C). The ratio over the freshly isolated cells confirmed the significant difference for pericytes and both EPC and mature EC. Indeed, compared to static condition with only 0.7- and 1.1-time difference, perfusion led to 4.5– and a 3.2–fold increase for pericytes and EC, respectively (Fig. 3B). Observing together all the single data collected from the six analyzed donors, perfusion showed a reproducible increase of the pericyte subpopulation for all the investigated cell primaries (Fig. 3C). Immunofluorescence staining for phospho-MAPK/ERK(1/2) (pERK(1/2)) highlighted the effects of the perfusion over this pathway, which is indeed one of the most SS-affected (Fig. 3D). Cells expressing both p44^ERK-1^ and p42^ERK-2^, using an antibody that recognizes their phosphorylated forms, significantly increased over time in perfusion compared to static culture (Fig. 3E).

Immunofluorescence staining for CD146, CD31 and pERK(1/2) revealed on the one hand that in both conditions, most of the cells expressing pERK(1/2) were neither endothelial cells (CD31^+^) nor pericytes (CD146^+^) (Supplementary Fig. S2A). On the other hand, immunofluorescence co-staining for CD90 and pERK(1/2) demonstrated that in both conditions the cells expressing pERK(1/2) were mainly of mesenchymal origin (CD90^+^) (Supplementary Fig. S2B).

### *In vivo* results

The *in vivo* angiogenic potential and host interaction of the engineered tissues were assessed in subcutaneous pockets of nude rats. This is considered a simpler model relatively to a disease one without any possible confounding effects (e.g. chronic inflammation). Although nude rats were used to avoid an immune response directed towards the implanted human cells, T-cell-deficient animal models still possess a normal innate immune response leading to a possible foreign body reaction to biomaterials^35,36^.

#### Perfusion accelerated *in vivo* vessel ingrowth

**Figure 4:**
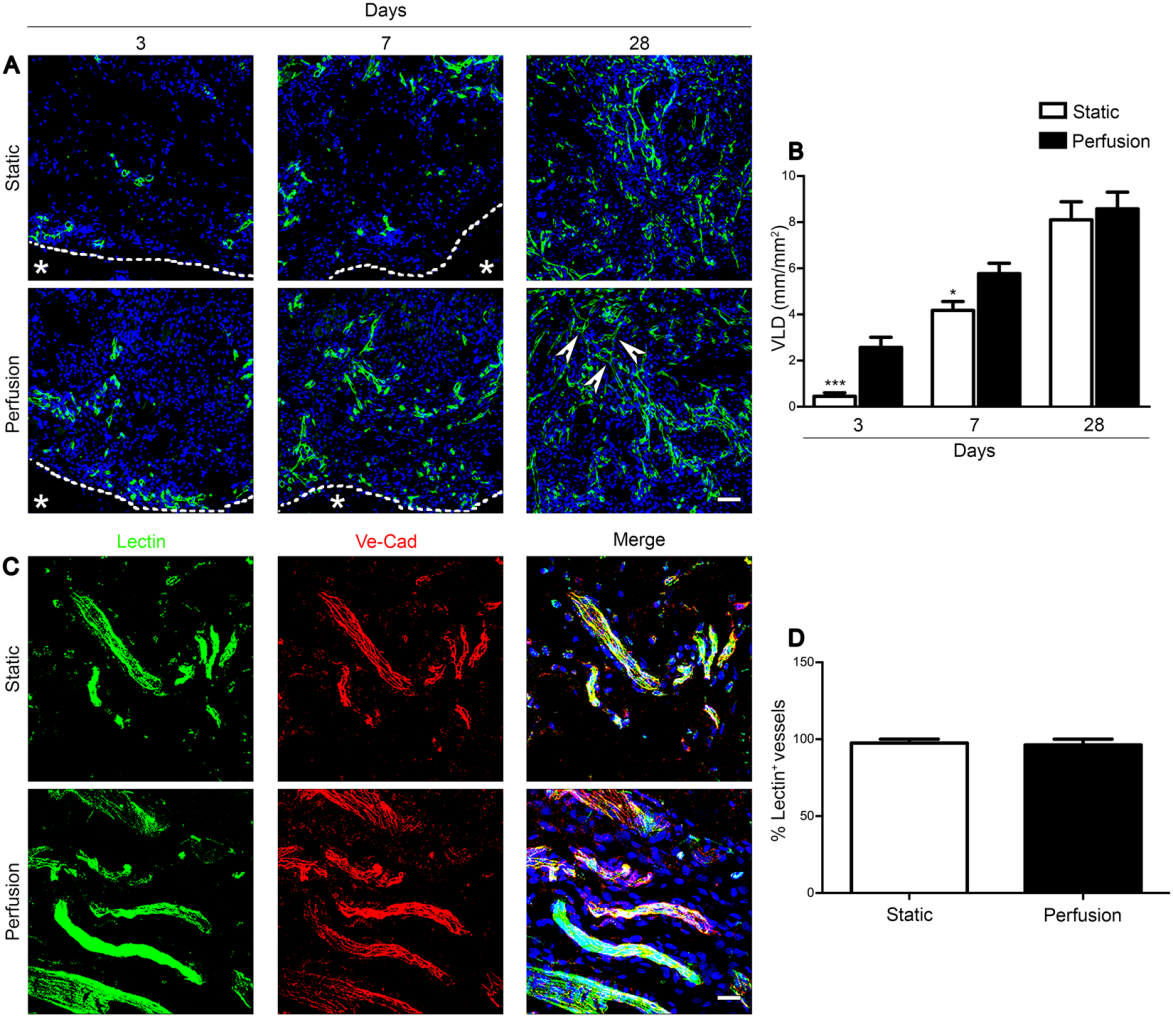
Perfusion accelerated *in vivo* vascularization of engineered tissues. (**A**) Representative immunofluorescence images stained for endothelial cells (CD31; green). Dashed lines outline the border between the patch and rat tissue (identified by the *). White arrows indicate branching points in perfusion condition at 28 days. Scale bar = 50 μm. (**B**) VLD quantification normalized over the analysed area. (**C**) Representative immunofluorescence images stained for EC (Ve-Cad; red) and intravascular marker (lectin; green) injected prior to sacrifice at 28 days. Nuclei were stained with DAPI (blue). Scale bar = 20 μm. (**D**) Percentage of the total vessels that resulted perfused by blood (lectin^+^) at the moment of sacrifice. *p < 0.05, ***p < 0.001. (n donor = 3).

In static constructs, the infiltrated vessels were rather few and limited to the border with the host tissue, whereas in the perfused patches, numerous capillaries, also in the center of the construct, were already observed after 3 days (Fig. 4A). At later time point, the vessel ingrowth greatly increased and was uniformly distributed for both conditions. The vessel length density (VLD) was significantly superior in perfused tissues at 3 and 7 days compared to static constructs (5.6- and 1.3-fold higher, respectively; Fig. 4B). Although no significant difference was found in the VLD at 28 days, branching points (Fig. 4A), that indicate the formation of a well inter-connected and physiological microvascular network, were observed almost exclusively in perfusion tissues. Moreover, the newly induced blood vessels resulted to be functionally connected to the host network. Indeed, co-localization of the green fluorescent signal (perfused circulating lectin) with the Ve-Cadherin (Ve-Cad) staining showed the generation of functional vessels preferentially close to the border with the host tissue at 3 and 7 days (Supplementary Fig. S3A) and uniformly distributed at 28 days (Fig. 4C). The majority of the newly formed vessels (>than 98%) was functional both at earlier (Supplementary Fig. S3B) and later time point (Fig. 4D).

#### Perfusion fostered a superior human cell engraftment *in vivo*

Implanted cells were detected both in static and perfusion constructs (Supplementary Fig. S4A,S4B). The human cell density was higher at all the three time points in perfusion compared to static culture with a statistically significant and biological relevant difference at 7 (151.2 ± 21.7 and 59.1 ± 7.2 cells/mm^2^, respectively) and 28 days (429.5 ± 38.1 and 342.3 ± 25.8 cells/mm2, respectively; Supplementary Fig. S4C). Proliferating human cells were observed in both conditions (Supplementary Fig. S4A,S4B); in perfusion, their number decreased with time *in vivo* remaining however always higher than static condition, with a significant difference at 7 days (21.5 ± 5.6 and 4.2 ± 1.2 cells/mm^2^, respectively; Supplementary Fig. S4D). Human apoptotic cells were also present in both conditions (Supplementary Fig. S5A,S5B). However, compared to static control, the dynamic condition showed a lower number of apoptotic human cells, with a significant difference at 3 (4.7 ± 0.8 and 2.2 ± 0.4 cells/mm^2^, respectively) and 28 days (12 ± 1.5 and 5.1 ± 1.4 cells/mm^2^, respectively; Supplementary Fig. S5C). Notably, the ratio between living (Human Nuclei specific –HuNu^+^) and apoptotic cells (HuNu^+^ and cleaved caspase-3^+^ - Casp-3^+^) demonstrated and confirmed the significantly superior engraftment of the perfusion-conditioned human cells at all the three time points (5.8 ± 1.8 vs 19.5 ± 1.6, 9.4 ± 1.7 vs 21.7 ± 0.2, and 29.7 ± 1.6 vs 82.2 ± 0.96 cells/mm^2^, respectively; Supplementary Fig. S4E).

#### Perfusion promoted *in vivo* the formation of human-origin blood vessels

**Figure 5:**
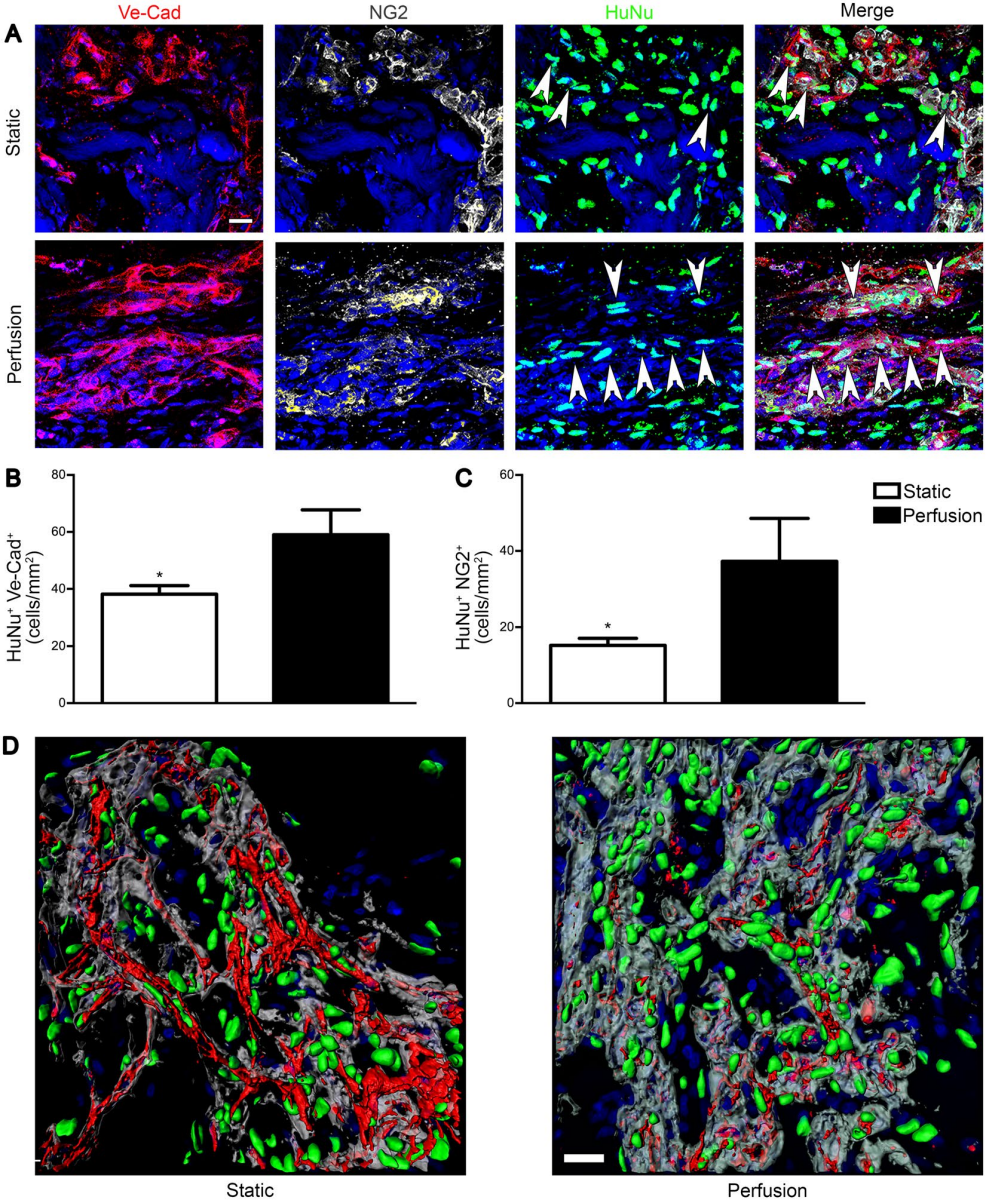
Perfusion-cultured human cells were directly involved in the formation of blood vessels. Patches were analysed after 28 days *in vivo*. (**A**) Representative immunofluorescence images of EC (Ve-Cad; red), pericytes (NG2; grey) and HuNu (green). White arrows indicate human cells directly involved in the formation of blood vessel Ve-Cad^+^ or NG2^+^. Quantification of the human cells Ve-Cad^+^ (**B**) and NG2^+^ (**C**) normalized over the analyzed area (n donor = 3). (**D**) 3D reconstruction of immunofluorescence images of EC (Ve-Cad; red), basal lamina (Laminin; grey) and HuNu (green). Nuclei were stained with DAPI (blue in **A**,**D**). *p < 0.05. Scale bar = 20 μm.

At early time points, only a few human cells were Ve-Cad^+^ or NG2^+^ (Supplementary Fig. S6A). In perfusion-cultured constructs, already at 3 days, human cells were actively taking part in the formation of some vascular structures, mainly as EC (Supplementary Fig. S6A), whereas only at day 7 human EC were present also in static constructs (Supplementary Fig. S6A). Quantification confirmed the overall low presence of human EC especially in the static condition at 3 days, in which no Ve-Cad^+^ cells were observed (Supplementary Fig. S6B). Remarkably, after 28 days perfusion culture supported the development of mature human-origin blood vessels, surrounded by human pericytes; whereas, in static constructs only a few implanted cells directly participated in the formation of new vascular structures (Fig. 5A). Human EC and NG2^+^ pericytes were significantly superior in perfusion-cultured constructs compared to the static condition (1.54- (Fig. 5B) and 2.4–times higher (Fig. 5C), respectively). Similarly, 3D reconstruction of the blood vessels corroborated the previously shown results: in static culture the majority of the implanted human cells resulted to be mainly close to, but not embedded in the basal lamina of the newly formed capillaries, whereas perfusion-culture led to human cells clearly surrounded by basal lamina at 28 days (Fig. 5D).

#### Pericytes modulated the factor release and the host foreign body response

Perfusion culture led to the development of an *in vitro* pro-angiogenic environment able to induce a rapid vascularization *in vivo*. We hypothesized that, among the beneficial angio-competent effects observed, the pericyte (CD146^+^) increase could represent the fundamental responsible for the obtained findings.

Depletion of CD146^+^ cells resulted in a high efficiency ( ≥ 90%) without affecting the other cell subpopulations.

**Figure 6:**
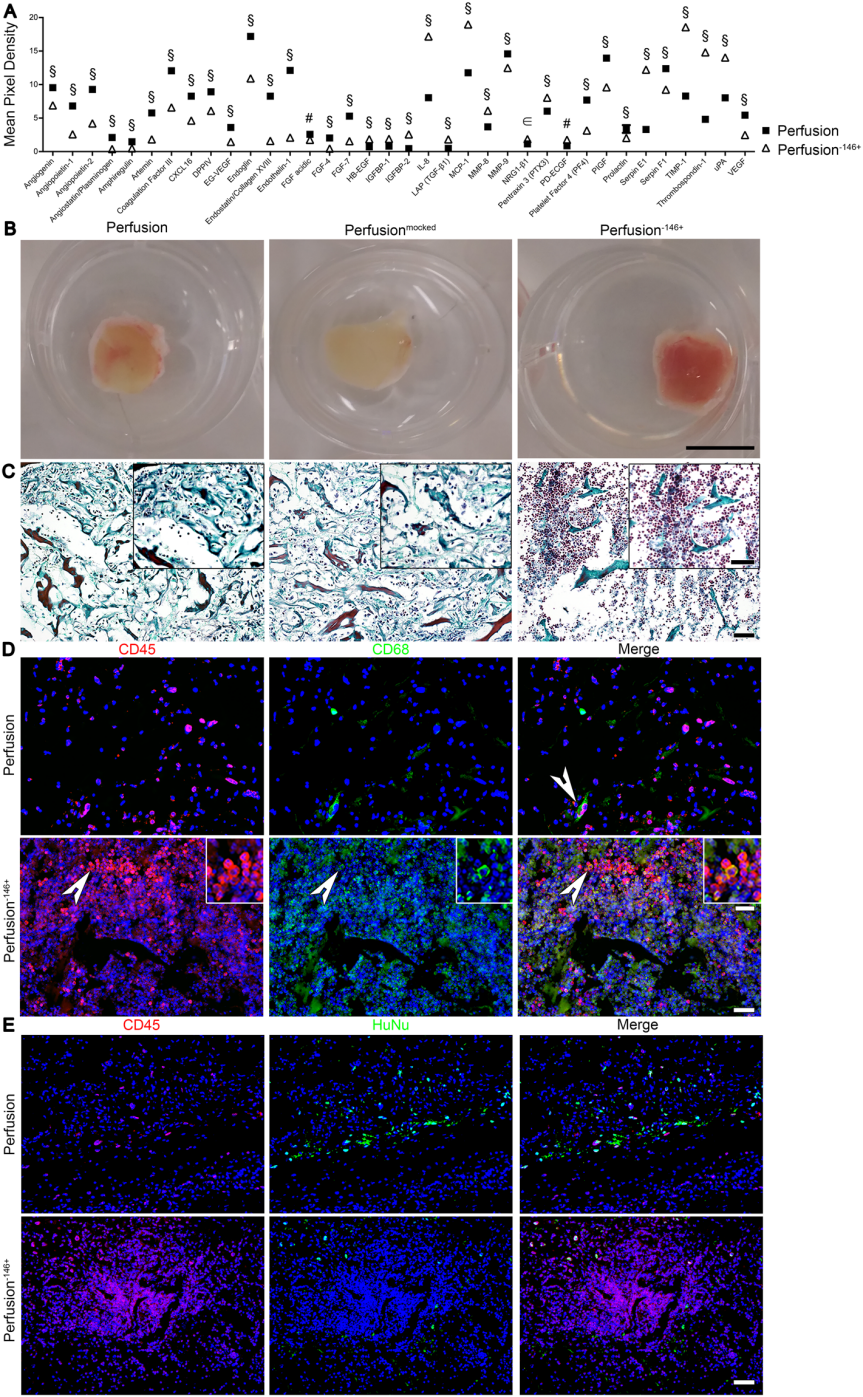
Pericytes modulated the factor release and increased host response. (**A**) Human angiogenesis proteome profiler array between perfusion and perfusion^−146+^ supernatants. Data are presented as mean pixel density normalized on DNA. § = *** p < 0.001; # = ** p < 0.01; ϵ = * p < 0.05 (n donor = 3). (**B**) Low magnification pictures for three different conditions: perfusion, perfusion^mocked^ (same technical procedure but no primary antibody), and perfusion^−146+^. 3 days *in vivo* time point. Scale bar = 1 cm. Representative images stained for Masson’s trichrome (**C**) and immunofluorescence images stained for hematopoietic cells (CD45; red) and macrophages (CD68; green) (**D**) and for hematopoietic cells (CD45, red) and human cells (HuNu, green) (**E**) of constructs generated in perfusion and perfusion^−146+^ conditions. Nuclei were stained with DAPI (blue). 3 days *in vivo* time point. Scale bar = 50 μm. White arrows indicate differentiated cells along a macrophage-lineage. Inset images in **C** and **D** bottom panels represent high magnification views with a scale bar = 12.5 μm.

The pericyte depletion led to a significantly different up-regulation of only 8 out of 25 pro-angiogenic factors compared to the 17 promoted by culturing the whole SVF population in perfusion. The major part of the down-regulated factors has a tight correlation with the pericytes as the coagulation factor III (TF), endoglin, MMP-9 and VEGF^37–40^. On the contrary, the pericyte depletion caused an up-regulation of the 55.5% anti-angiogenic factors (5 out of 9), as well as for all the immunomodulatory cytokines (IL-1β, IL-8, leptin, MCP-1, and MIP-1α; Fig. 6A), suggesting a possible role in the host foreign body reaction. After 3 days *in vivo*, a macroscopic analysis showed indeed that the explanted engineered tissues generated by pericyte-depleted SVF were entirely enclosed in a thick highly vascularized connective tissue capsule (Fig. 6B). This response was present only to a limited extent in the controls (whole fresh or mocked-sorted SVF based samples), therefore excluding a possible negative effect of the sorting procedure on the cells. The detected reaction could be instead correlated with the lack of pericytes. The implants composed by pericyte-depleted SVF cells showed indeed a typical foreign body reaction. An inflammatory infiltrate dominated by neutrophilic granulocytes was observed throughout the entire cross-section (Fig. 6C). Constructs generated by the whole SVF cells instead showed reduced inflammatory cell infiltration confined at the border with the host tissue. Numerous hematopoietic cells (CD45^+^) were observed within the entire implant when pericyte-depleted SVF cells were used (Fig. 6D), and a large amount of them differentiated along a macrophage lineage as shown by the co-localization of CD45^+^ and CD68^+^ markers (Fig. 6D). In contrast, perfusion-based constructs generated with the whole SVF showed only a scattered infiltration of hematopoietic cells upon implantation. Moreover, immunofluorescence co-staining for CD45 and HuNu showed that the major part of the infiltrating cells derived originally from the host and not differentiated from human SVF cells (Fig. 6E).

#### The depletion of pericytes inhibited the *in vitro* pre- and *in vivo* early vascularization

**Figure 7:**
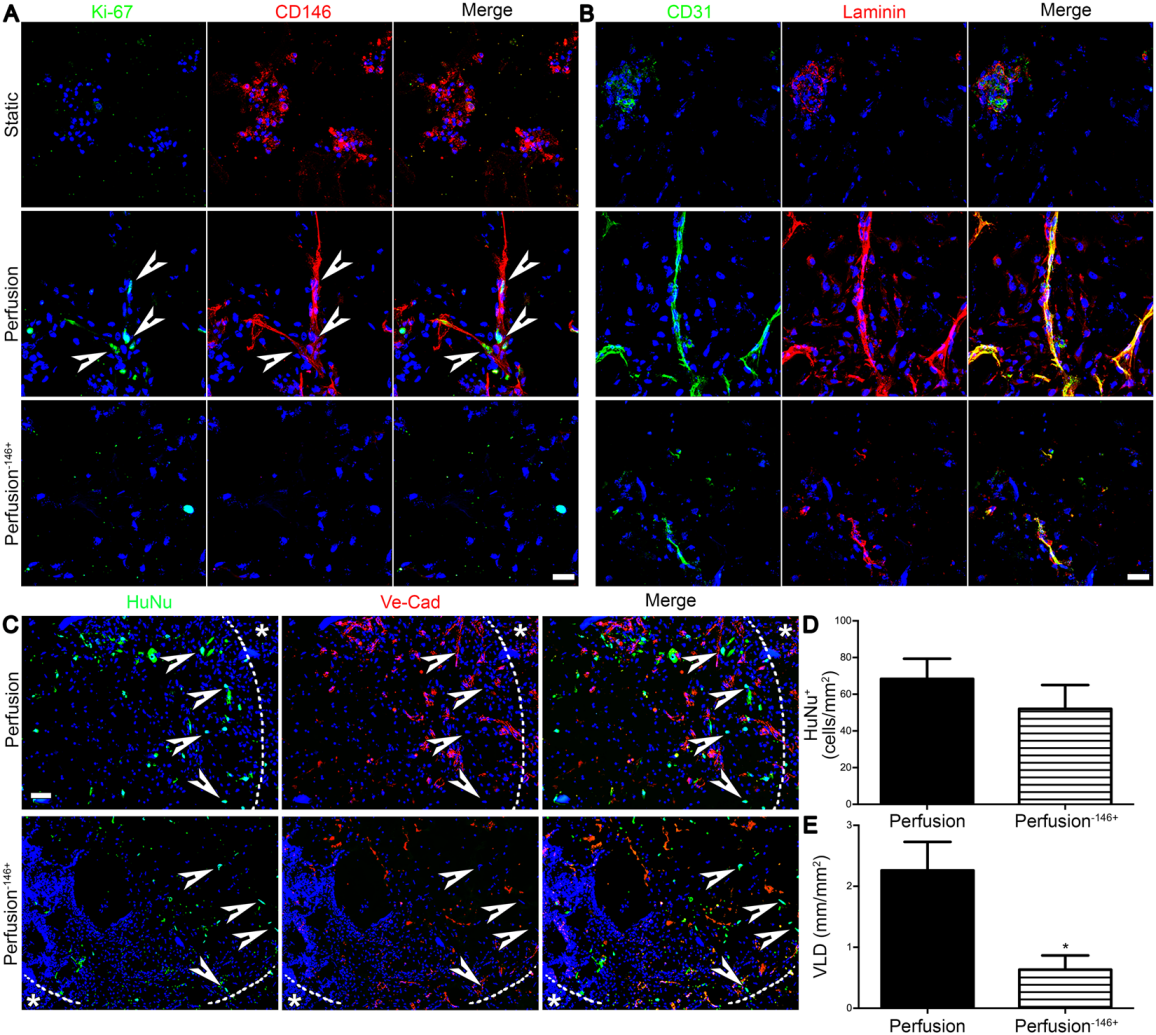
Pericyte depletion decreased cell proliferation and *in vitro* pre- and *in vivo* early vascularization. Patches were analysed after 3 days *in vivo*. (**A**) Representative *in vitro* immunofluorescence images of proliferating cells (Ki-67; green) and pericytes (CD146; red). White arrows indicate proliferating CD146^+^ cells. Scale bar = 20 μm. (**B**) Representative *in vitro* immunofluorescence images for EC (CD31; green) and basal lamina (laminin; red). Scale bar = 20 μm. (**C**) Representative *in vivo* immunofluorescence for human cells (HuNu; green) and EC (Ve-Cad; red). Nuclei were stained with DAPI (blue in **A**–**C**). Dashed line outlines the border between the patch and the rat tissue (identified by the *). White arrows indicate human cells. Scale bar = 50 μm. (**D**) Quantification for the HuNu^+^ cells normalized over the analysed area. (**E**) VLD quantification normalized over the analysed area. *P < 0.05 (n donor = 3).

The proliferation of pericyte-depleted SVF was remarkably reduced compared to the perfusion of the whole SVF, similarly to what was previously observed in the static condition (Fig. 2D,E). Within the entire SVF cell population, perfusion stimulated preferentially the proliferation of the CD146^+^ cells (Fig. 7A), further corroborating the outcome of the flow cytometry analysis displayed in Fig. 3A. Perfusion^−146+^ led instead to a lower proliferation of cells negatively stained for the CD146. *In vitro* staining for the endothelium (CD31) and basal lamina (laminin) confirmed the formation of only cell clusters in the static condition and the development of more elongated vessel-like structures under perfusion. However, even if all the three conditions showed the presence of cells both CD31^+^ and laminin^+^, only the tissues cultured under perfusion with the pericyte-enriched SVF population demonstrated the formation of complex elongated endothelial structures covered by the basal lamina (Fig. 7B).

Three days after implantation, human implanted cells were equally present in both the perfusion-based constructs (Fig. 7C), as corroborated by their quantification (68.3 ± 11.1 and 52.1 ± 12.9 cells/mm^2^, respectively; Fig. 7D). Nevertheless, perfusion^−146+^ led to a scattered generation of short vessels mainly at the border with the host tissue (Fig. 7C), whereas perfusion-based SVF enhanced the formation of complex and elongated vascular structures (the VLD was 2.7-fold superior; Fig. 7E).

## Discussion

In this study, we demonstrated the key role of perfusion-generated hydrodynamic-based SS in reproducibly engineering an *in vitro* environment, strongly promoting the growth of pericytes, the formation of cord-like structures, and the release of pro-angiogenic factors. We also showed that this engineered niche efficiently led *in vivo* to a prompt and functional angiogenesis, an enhanced engraftment of the implanted cells, and to a reduced host foreign body response. Based on these key findings and the *in vivo* functionality, we here define the perfusion-cultured construct as an *in vitro* engineered *angiogenic niche*, demonstrating the crucial role of perfusion in pericyte enrichment and in generating and maintaining this specialized microenvironment.

Due to the morphological localization and the strong connection with EC, pericytes are indirectly influenced by the wall SS acting on the endothelium^41^. Human capillary with a diameter between 6 and 12 μm have shown an SS ranging between ≈21 and 105 dyne/cm^2^ ^42^. Based on the study by Cioffi *et al*.^43^ conducted in the same bioreactor with a comparable sponge in terms of pore architecture and size, the average SS generated by our perfusion system was ≈0.63 dyne/cm^2^. However, high flow rates ranging 5–22 mL/min to resemble a capillary-like SS would not conform to our in-development vascular structures and could most likely harm cells and impede their adhesion to the scaffold. SS are often associated with signaling cascades in EC, activating transcriptional genes, including platelet-derived growth factor-B and transforming growth factor, and mediation of pericyte recruitment and proliferation^27,41^. Moreover, also the classical MAPK/ERK(1/2) signaling pathway participates in cellular mechanotransduction, specifically, by ‘translating’ mechanical signals, as SS, into intracellular biological signals that regulate and control major cell functions, such as proliferation, differentiation, and migration^44–47^. Several studies have indeed demonstrated not only that the presence of pericytes in co-culture with EC induced an increase in the endothelial phosphorylated forms of p42/p44 MAPK/ERK(1/2), but also the critical roles of the ERK1/2 pathway in promoting pericyte proliferation^48,49^. However, besides the correlation between the ERK1/2 pathway and the EC/pericytes, it has also been shown that the SS causes a strong ERK1/2 activation also in human MSC both cultured in 2D^50^ and in 3D^51^, corroborating the results of the present study, in which ERK1/2 activation occurred mainly in human MSC exposed to SS. Therefore, based on these findings we can speculate that the SS acting on the heterogeneous SVF population activates the downstream ERK1/2 pathway in the most abundant MSC population, which then help in recreating and modulating the favorable surrounding angiogenic environment and most likely supporting the pericyte proliferation.

The identification of pericytes in tissue is a complex process because there is not a single marker and only their morphology and position in the vessel are the real distinguishing hallmarks. Consistent with Corselli *et al*.^18^, in our study pericytes derived from adipose tissue have been identified by flow cytometry as CD45^−^ CD34^−^ CD146^+^. However, when we have performed the depletion only single CD146^+^ cells have been taken into account, considering a more general perivascular cell population, rather that a pure pericyte-portion. Indeed, CD146^+^ cells together with CD34^+^ cells have been described as both a rare population of adipose-derived pericytes^40^ and a pericyte subset that may be transitional between pericytes and supra-adventitial adipose stromal cells (CD34^+^ CD31^−^ CD146^−^), sharing the same anatomical location^52^. In our study, among the 12.69 ± 2.12% of the CD45^−^/CD146^+^ cells, the CD34^+^/CD146^+^ subset represents the 13.3% (1.7 ± 0.5%) before the culture, and the 8% after the perfusion respect to the 91.9% of the CD45^−^CD34^−^CD146^+^ pericytes. We, therefore, consider the possible role of CD45^−^/CD34^+^/CD146^+^ in this experimental setup less crucial compared to the pericytes defined as CD45^−^/CD34^−^/CD146^+^.

The lack of CD146^+^ pericytes observed in the perfusion^−146+^ condition led to a remarkable inflammatory cell invasion. Several studies suggest that, in addition to the usual neutrophils and mononuclear cells (lymphocytes and monocytes), even pericytes contribute to suppression and resolution of the inflammatory reaction ascribed to multiple mechanisms involving paracrine effects and cellular interactions^53,54^. However, since in our model we did not observe yet many human-originated pericyte-coated vessels at an early time point, we hypothesized that the main pericyte contribution to the immune-suppression was paracrine-modulated. Indeed, in the cell depleted-condition, all the immunomodulatory proteins present in the array were up-regulated compared to the pericyte-enriched condition. Confirming our results, Chen *et al*.^54^ demonstrated that pericytes contributed to reducing the infiltration of host phagocytic cells within a murine ischemic myocardium, mainly thanks to the expression of a considerable array of anti-inflammatory cytokines.

In this study, we described for the first time the possibility to reproducibly engineer *in vitro* functional *angiogenic niches* by 3D perfusion-based SVF cell culture. Our engineered *angiogenic niche* would represent an optimal model for investigating fundamental tissue/organ regeneration mechanisms, revealing complex interactions between the angiogenic and other stem cell niches or tissue-specific parenchymal cell populations. In addition, the proposed angiogenic environment suggests a successful strategy for vascularizing mm-thick engineered tissue by simply harnessing naturally angio-competent factors, such as highly vasculogenic cells and hydrodynamic-based SS. The here proposed approach ensures circumventing the difficulties associated with the complex research of the needed balance/homeostasis among the main vascular building blocks. The resulting outcomes will foster the transition of our *angiogenic niche* toward a clinical setting thanks to the use of ready-to-use autologous cells. All tissues potentially affected by ischemic events (e.g. limb muscle and myocardium) that urgently need for an adjuvant angiogenic therapy for inducing rapid vascularization, therefore guaranteeing cell survival and engraftment, would benefit from our developed angiogenic niche.

## Materials and Methods

### Cell Preparation and Perfusion-Based Culture

#### Stromal Vascular Fraction Cell Isolation

Liposuctions were obtained from nine healthy donors undergoing plastic surgery after informed consent and according to a protocol approved by the Ethical Committee of Basel University Hospital. All investigations conform to the declaration of Helsinki. The adipose solution was digested with 0.075% w/v collagenase type II (Worthington Biochemical Corporation) in phosphate buffered saline (PBS, Invitrogen) at 37 °C undergoing continuous shaking for 60 min. After centrifugation at 1,500 rpm for 10 min, the floating lipid-rich layer was discarded and the cellular pellet was washed once with PBS. Cell suspension was strained through a 100 µm followed by a 70 µm nylon-mesh in order to remove fibrous debris. The resulting SVF cells were then re-suspended in growth medium consisting of DMEM high glucose (Sigma–Aldrich), 10% v/v fetal bovine serum (FBS, HyClone), 1% v/v penicillin/streptomycin, 1% v/v glutamine, and 1% v/v hepes (all from Sigma-Aldrich). Nucleated cells stained with crystal violet (Sigma–Aldrich) were counted in a Neubauer chamber. Freshly isolated SVF cells were frozen in 10% v/v dimethyl sulfoxide (DMSO), 90% v/v FBS medium and stored in liquid nitrogen. For experiments, SVF cells were quickly thawed in a 37 °C water bath and immediately used. Cell survival rate was between 70% and 90%.

#### Perfusion-Based Bioreactor Culture System

For the direct perfusion of a cell suspension through the pores of 3D scaffolds, a previously developed bioreactor was used (Cellec Biotek AG). Discs (12 mm diameter, 3 mm thickness) of type I collagen (Ultrafoam^®^ collagen hemostat from Davol, Inc.) were hydrated in culture medium (24 h at 37 °C). The scaffold was then placed between two silicon O-rings that were holding it in place and allowed the direct perfusion of the medium through only the inner 8 mm diameter of the collagen sponge^55,56^.

#### Perfusion-Based Cell Seeding and Culture

SVF cells were seeded at 14.6 × 10^6^ cells/cm^3^, corresponding to 2.2 × 10^6^ cells/scaffold, with a bidirectional flow rate of 3 mL/min for 18 h using a syringe pump (Programmable Harvard Apparatus PHD ULTRATM 2000), as described in previous studies^55,56^. The cell-seeded constructs were then cultured in 8 mL growth medium for additional 5 days either with a unidirectional perfusion flow rate of 3 mL/min using a peristaltic pump (Ismatec SA, Reglo Digital MS-4/8) or in static condition as control. Culture was performed within incubator at 37 °C and 5% CO_2_ and the medium was not changed during the entire culture time.

### Animal studies

Animals were treated in compliance with the Swiss Federal guidelines for animal welfare and all procedures were approved by the Veterinary Office of the Canton Bern (Bern, Switzerland) and conform to the Directive 2010/63/EU of the European Parliament.

Eight-week-old male nude rat (Hsd: RH-rnu/rnu, Envigo, weight range 250 ± 16 g) were anesthetized by inhalation using a mixture of oxygen (0.6 L/min) and isoflurane (1.5–3 vol %). Four grafts were implanted in distinct subcutaneous pockets in the back of each rat (4 samples per experimental groups for three different donors). At the moment of sacrifice after 3, 7 and 28 days post-implantation, the total rat vasculature was perfused with 1% w/v paraformaldeide (PFA) following anesthesia by intraperitoneal injection of a mixture of ketamine (100 mg/Kg) and xylazin (10 mg/Kg). The harvested constructs were further fixed in PFA 1% w/v for 30 min and leaved in sucrose 30% w/v overnight at 4 °C before embedding in OCT compound (CellPath), and frozen in isopentane vapors cooled in liquid nitrogen. In some experiments, fluorescein isothiocyanate (FITC)-labeled Lycopersicon esculentum lectin (250 μg in 250 μl; Vector Laboratories) was injected into the femoral vein and allowed to circulate for 4 min before sacrifice in order to label perfused vessels as previously described^57^.

### Assessments

#### DNA quantification

DNA quantification was performed as previously described^58^. Briefly, one half of each construct was weighed and digested overnight at 57 °C in proteinase K solution (1 mg/mL proteinase K, 50 mM TRIS, 1 mM EDTA, 1 mM iodoacetamide, and 10 mg/mL pepstatin-A; Sigma–Aldrich) in double distilled water or potassium phosphate buffer. DNA quantification was performed by using a fluorescence based kit, namely CyQUANT^®^ Cell Proliferation Assay (Invitrogen), according to the manufacturer’s protocol. The analyses were carried out measuring fluorescence with a SpectraMax Gemini XS Microplate Spectrofuorometer (Molecular Devices). Excitation and emission wavelengths were 485 and 583 nm, respectively. Three samples were tested for each donor (n donor = 3) for all the experimental groups.

#### Histological Evaluation

All histological analyses have been performed on 10 µm thick frozen sections. For basic histomorphological evaluation, sections were stained with hematoxylin and eosin (H&E), Picro-Sirius Red, and Masson’s Trichrome (MT) according to the manufacturer’s protocol. For immunofluorescence analyses cryosections were incubated for 1 h in 0.3% v/v Triton X-100 and 2% v/v normal goat serum, or 5% v/v donkey serum in PBS (blocking buffer), and then for 1 h in the following primary antibodies: (i) mouse anti-PECAM-1 (CD31,LubioScience), (ii) rabbit anti-Ki67 (Abcam), (iii) mouse monoclonal anti-Human Nuclei (HuNu) (clone 235-1, Millipore), (iv) rabbit anti-NG2 (Millipore), (v) rabbit anti-cleaved caspase3 (Cell Signaling), (vi) goat anti-Ve-Cadherin (Santa Cruz), (vii) rabbit anti-laminin (Abcam), (viii) rabbit anti-CD34 (Abcam), (ix) goat anti-CTGF (Santa Cruz), (x) mouse anti-CD146 (Abcam), (xi) rabbit anti-CD45 (Abcam), (xii) mouse anti-CD68 (Abcam), (xiii) rabbit anti-phospho-p44/42 MAPK (pErk1/2) (Cell Signaling), and (xiv) mouse anti-CD90 (Abcam). All antibodies were diluted at 1:100, except anti-NG2 at 1:200, anti-laminin at 1:400, and anti-pErk1/2 at 1:200 following methanol permeabilization.

Subsequently, tissue sections were incubated in dark for 1 h in fluorescently labeled Alexa488, Alexa546, or Alexa647 (Invitrogen) secondary antibody (dilution 1:200). Nuclei were stained using 4’,6-diamidino-2-phenylindole (DAPI, Invitrogen). All first and second antibodies were diluted in the blocking buffer. Fluorescence images were acquired with Olympus BX63 microscope (Olympus) or Zeiss LSM710 confocal microscope (Zeiss). Single-blind analyses were conducted during the *in vitro* and *in vivo* experiment.

#### Flow Cytometry

The SVF cell phenotype was determined by 6-channel cytofluorimetric analysis before and after the culture. For this latter condition, constructs were digested in 0.15% w/v collagenase (Worthington Biochemical Corporation) and cells were collected by centrifugation. Cells in suspension were then incubated for 30 min on ice with different fluorochrome-conjugated antibodies to human CD90 FITC, CD73 APC, CD31 FITC, CD34 APC-Cy7, CD146 PE, CD45 BV605, VEGFR2 PE, and CD11b APC (all from Biolegend) in staining buffer (PBS, 0.5% v/v FBS, 2 mM EDTA). 5 µl per million cells were used for all the antibodies according to the manufacturer’s protocol, and data were acquired with LSRFortessa^TM^ flow cytometer (BD Biosciences), analyzed using Flowjo v10.1r5 software (Tree Star), and presented as percentage over the live cells (DAPI positive) or ratio over the fresh SVF population.

#### Cell sorting

SVF cells freshly isolated as previously described underwent depletion of the pericyte components using the EasySep PE selection kit and an EasySep magnet (StemCell Technologies) according to the manufacturer’s protocol. Total SVF were depleted of CD146^+^ cells by positive selection following incubation with PE-CD146 antibody (Biolegend). The resulting SVF/CD146^−^ cellular fraction was used for the experiments. Mock cells underwent the same sorting procedure but without the primary antibody and were used as control.

#### Angiogenesis Arrays

To screen for angiogenesis-related proteins released in the supernatant, a human angiogenesis proteome profiler antibody array was used, according to the manufacturer’s instructions (R&D Systems, Inc.). Supernatants (7.7 ± 0.2 mL) were collected through the injection site for the perfusion condition and from the well for the static one. Signals were visualized using ImageJ 1.47 software (Research Service Branch, NIH). The array data were normalized to the background and to the DNA amount and quantified by measuring the sum of the intensities of the pixels within the spot boundary pixel area. Two samples were tested for each donor (n donor = 2) for all the experimental groups.

#### *In vitro* assessment of VEGF release

After 5 days of *in vitro* culture, supernatants (7.7 ± 0.2 mL) were collected as previously described and the concentration of released human VEGF was measured by ELISA kit (hVEGF, R&D Systems) according to manufacturer’s instructions. Three donors were assessed in triplicate for each experimental group. Data are expressed as pg of protein normalized to the total amount of DNA for each relative construct.

#### Image Analysis

All image analyses were performed using ImageJ 1.47 software (Research Service Branch, NIH) or CellSens software (Soft Imaging System) on at least triplicate samples per donor (n donors = 3), and ten representative fields (acquired by a 20x objective) were analyzed per constructs. For the *in vitro* engineered-tissue characterization, the overall cell proliferation rate was calculated as the percentage of the Ki-67-positive nuclei number versus the total number of cells. The amount and the complexity of the endothelial cord-like structures were quantified counting the number of aligned CD31 positive cells normalized to the total amount of CD31 positive cells. The expression of pERK1/2 was calculated as the percentage of the p(ERK1/2)-positive nuclei number versus the total number of cells after respectively 1 day of seeding, 3 and 6 days of culture in order to investigate the effect of perfusion over time. In the *in vivo* experiments, the VLD was determined by normalizing the length of CD31 positive vessels to the total area of each image field. The human nuclei were quantified by counting the HuNu positive cells to the total area of each image field. All the other *in vivo* quantifications (Ki-67-, cleaved caspase-3-, Ve-Cadherin- and NG2- positive cells) were similarly calculated considering as positive co-localizing the staining of interest with the HuNu to the total area of each image field.

#### Statistical Analysis

Data are expressed as mean ± standard error of the mean (SEM). Before statistical testing, Kolmogorov-Smirnov test was performed on all data sets to assess normal distribution. The data, which satisfied the normality test, were analyzed using a 2-tailed unpaired Student’s t test for all single comparisons and analysis of variance one-way ANOVA by for multiple comparison. Otherwise, non-parametric Mann-Whitney test for single comparison or Kruskal-Wallis test for multiple comparisons and Dunn’s post-hoc test were used. Results were considered to be statistically significant at p values < 0.05 (*p < 0.05, **p < 0.01, ***p < 0.001). The data were processed with GraphPad Prism 5 Software (GraphPad).

### Availability of materials and data

The authors will make materials, data and associated protocols promptly available to readers if requested.

## Electronic supplementary material

Supplementary Information
