# Supplementary material for: Engineering of an angiogenic niche by perfusion culture of adipose-derived stromal vascular fraction cells
Source: Sci Rep. 2017 Oct 27;7:14252. doi: 10.1038/s41598-017-13882-3 (PMC5660248; doi:10.1038/s41598-017-13882-3)
Supplement: Supplementary file 1 — Supplementary Information [file 41598_2017_13882_MOESM1_ESM.pdf]

Engineering of an *angiogenic niche* by perfusion culture of adipose-derived stromal vascular fraction cells

Giulia Cerino, Emanuele Gaudiello, Manuele Giuseppe Muraro, Friedrich Eckstein, Ivan Martin, Arnaud Scherberich, Anna Marsano

Supplementary Material

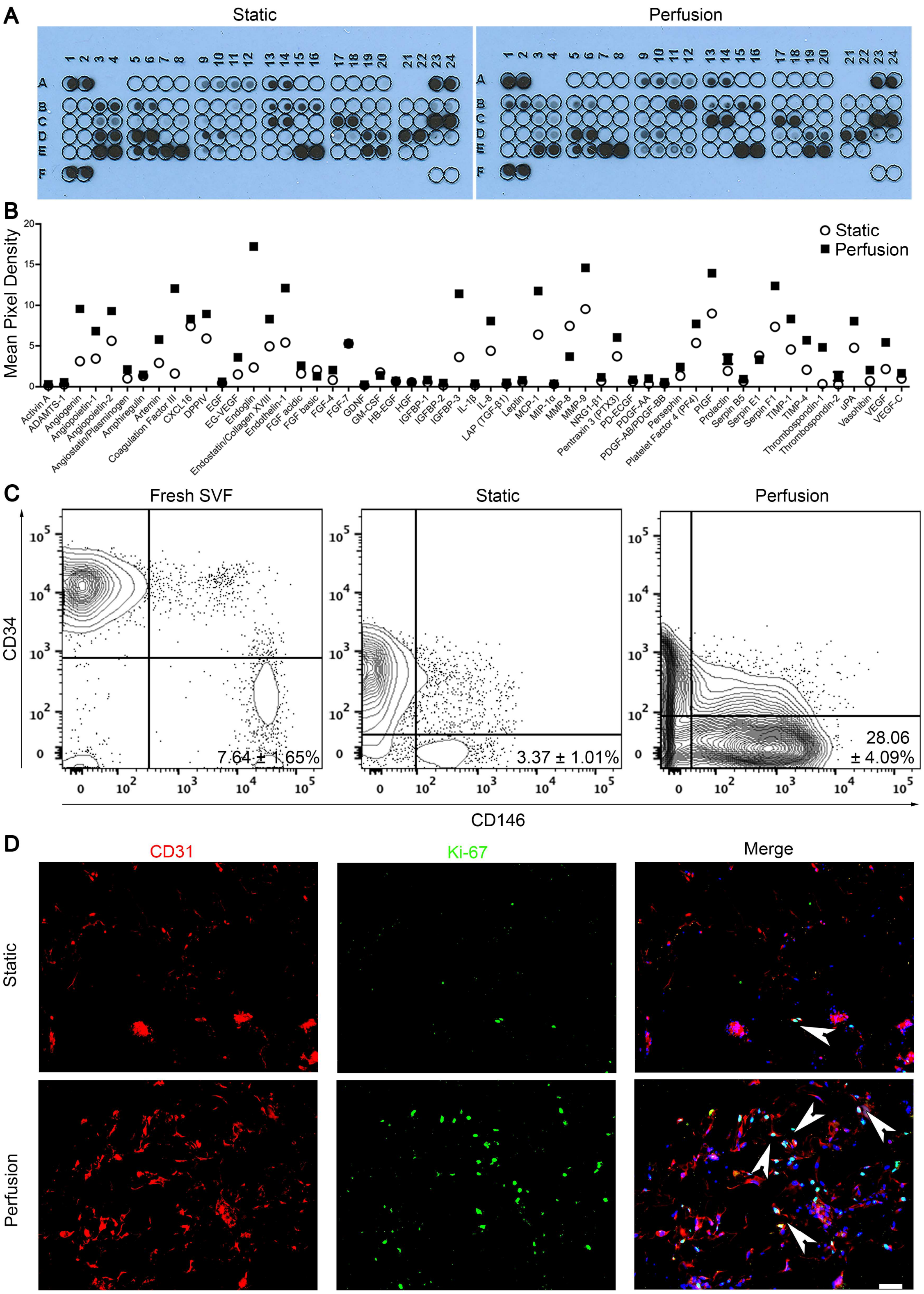

Supplementary Figure S1. Perfusion enhanced the SVF pro-angiogenic factor releasing profile. (**A**) Representative membrane scans. (**B**) The entire human angiogenesis proteome profiler array was assessed in the supernatants collected following 5-day culture in static or perfusion condition. Data are presented as mean pixel density normalized to the total amount of DNA of each relative construct (n donor = 3). (**C**) Representative contour plots for pericytes. (**D**) Representative immunofluorescence images for endothelial cells

(CD31, red) and cell proliferative marker (Ki-67, green). Nuclei were stained with DAPI (blue). Scale bar = 50 μm.

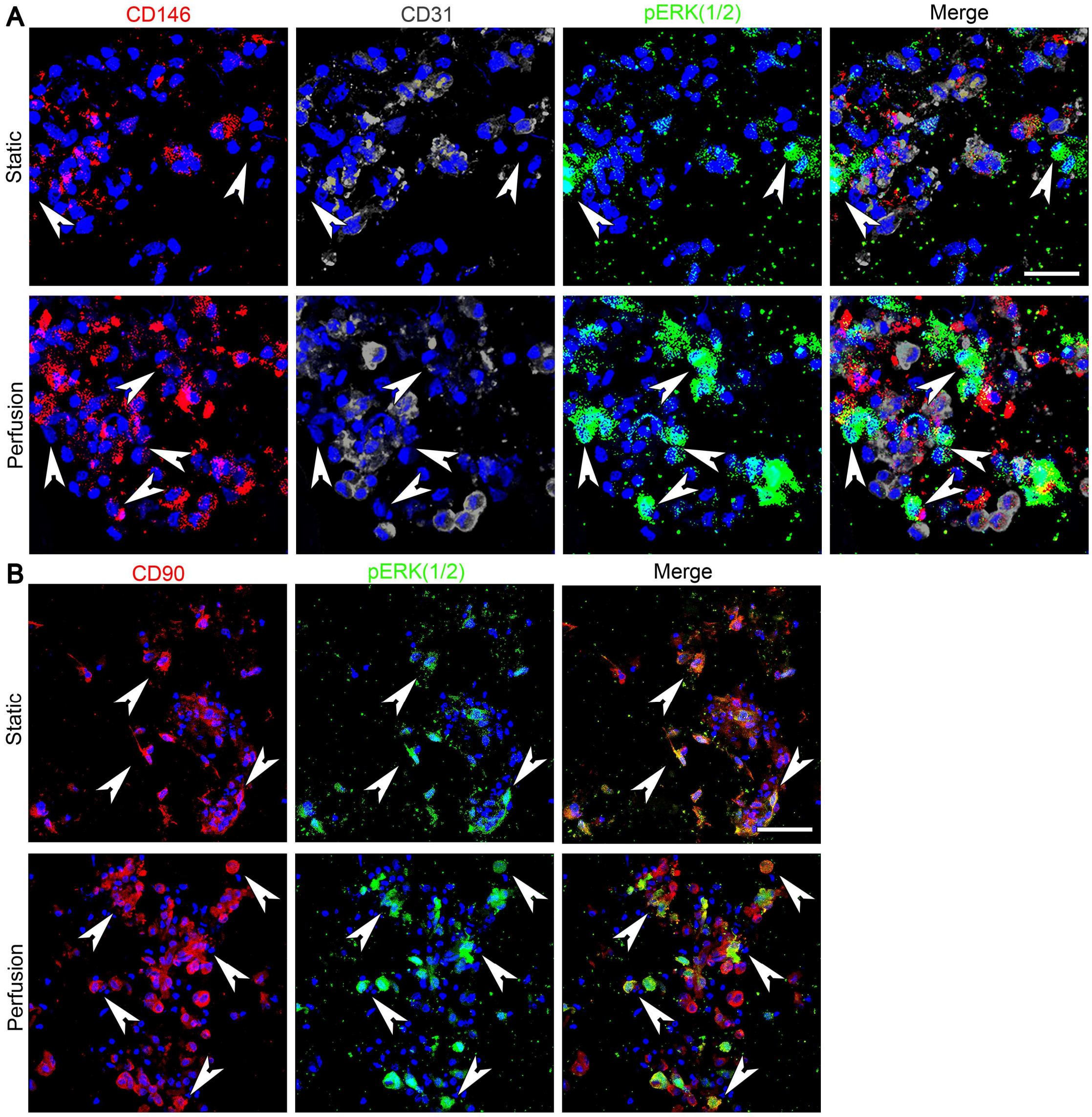

Supplementary Figure S2. Perfusion enhanced the expression of pERK1/2. (**A**) Representative immunofluorescence images for pericytes (CD146; red), endothelial cells (CD31; grey) and pERK(1/2) (green). (**B**) Representative immunofluorescence images for mesenchymal cells (CD90; red) and pERK(1/2) (green). Nuclei were stained with DAPI (blue). Scale bar = 20 μm.

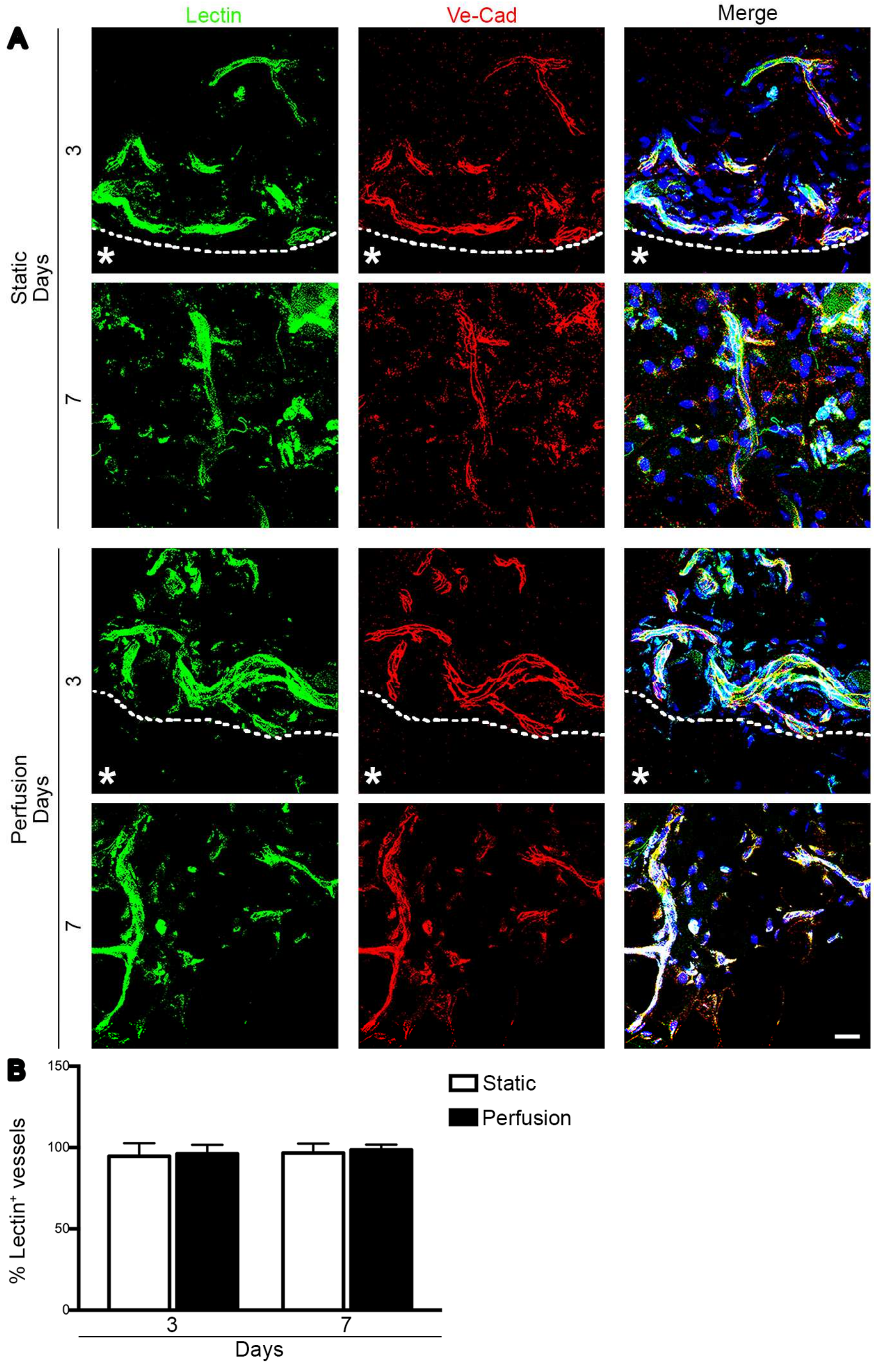

Supplementary Figure S3. Vessels observed at early time point were functionally connected to the host main circulation. (**A**) Representative immunofluorescence images for endothelial junctions (Ve-Cad; red) and intravascular marker injected prior sacrifice (lectin; green). Nuclei were stained with DAPI (blue). Dashed line outlines the border between patch and the rat tissue (identified by the *). (**B**) Quantification of % of the total vessels that resulted perfused by blood (lectin^{+}) at the moment of sacrifice (n donor = 3). Scale bar = 20 μm.

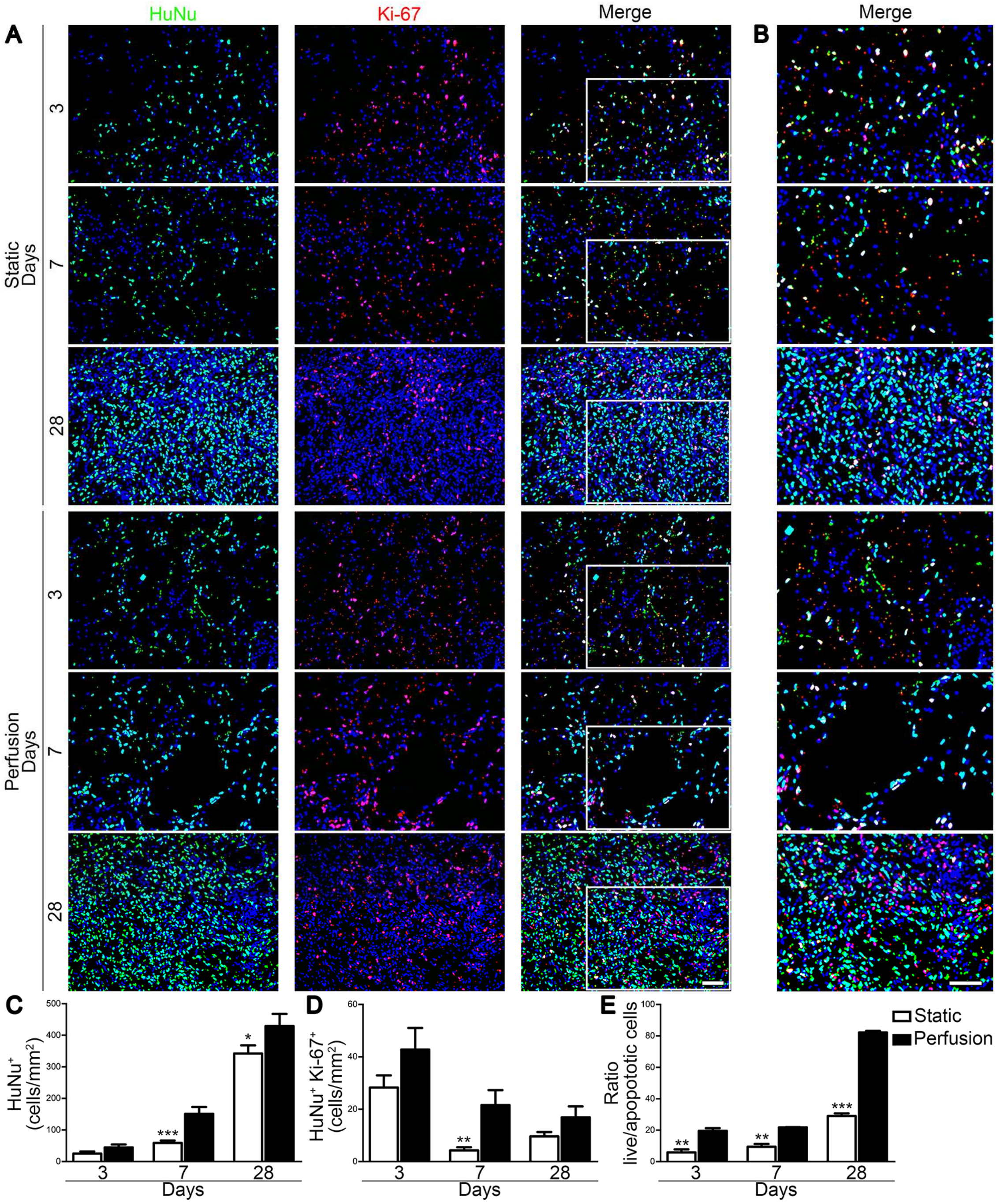

Supplementary Figure S4. Perfusion improved human cell engraftment upon implantation. (**A**) Representative immunofluorescence images of human nuclei (HuNu; green) and proliferating cells (Ki-67; red). Nuclei were stained with DAPI (blue). (**B**) Higher magnification immunofluorescence images of the area in the white rectangle in panel A. In white, cells Ki-67^{+} and HuNu^{+}. (**C**) Quantification of the human cell density. (**D**) Quantification of the human proliferating cells. (**E**) Quantification of the ratio of living cells (HuNu^{+}) and cells in apoptosis (cleaved caspase-3, Casp-3^{+}). *p<0.05, **p<0.01, ***p<0.001 (n donor = 3). Scale bar = 50 μm.

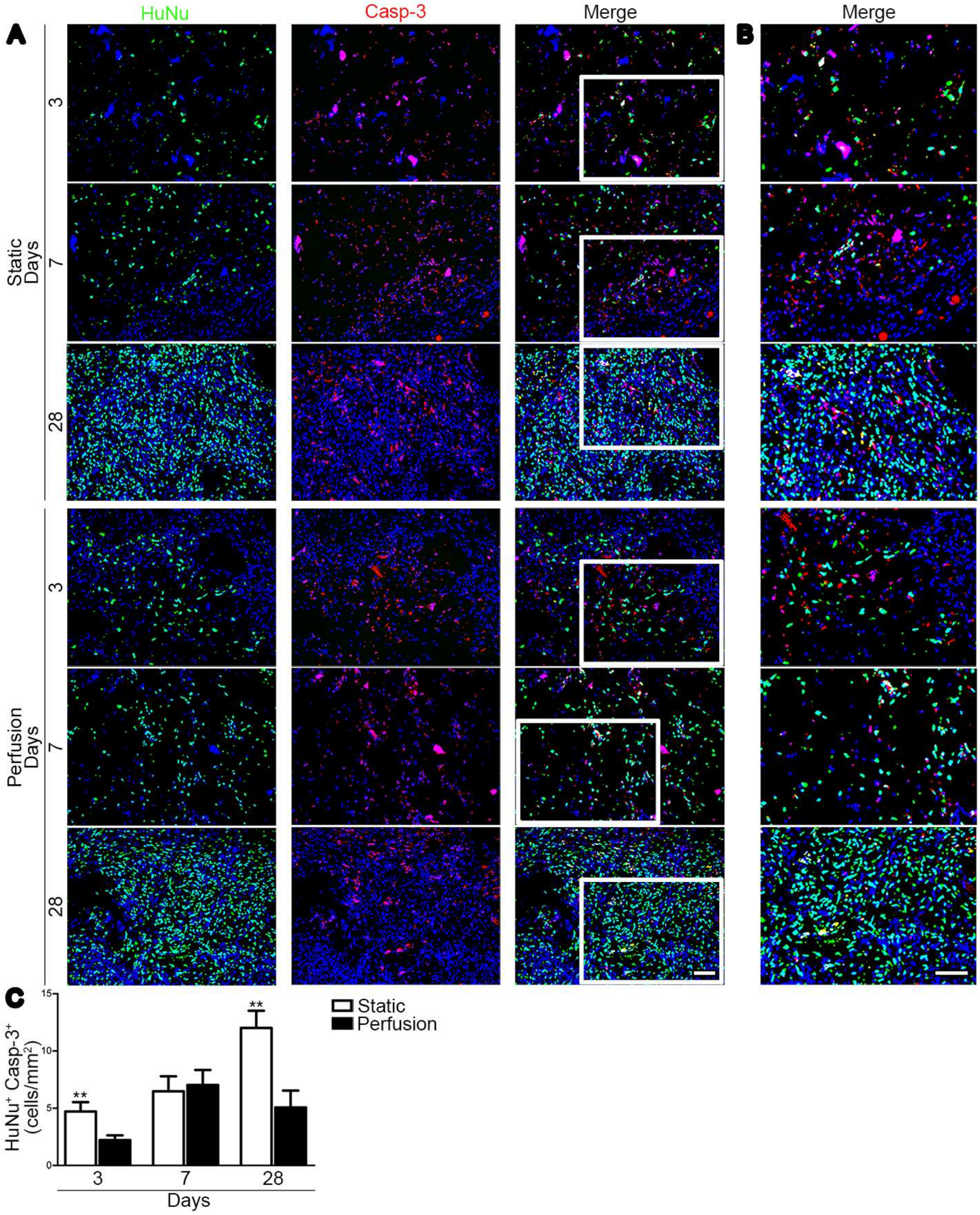

Supplementary Figure S5. Perfusion reduced the number of apoptotic implanted human cells. (**A**) Representative immunofluorescence images for implanted human cells (HuNu; green) and apoptotic cells (Cleaved Casp-3; red). Nuclei were stained with DAPI (blue). (**B**) Higher magnification immunofluorescence images of the area in the white rectangle in panel A. In white cells cleaved Casp-3$^+$ and HuNu$^+$. (**C**) Quantification of the apoptotic human cells cleaved Casp-3$^+$ normalized over the analysed area. **p<0.01. (n dono r= 3). Scale bar = 50 µm.

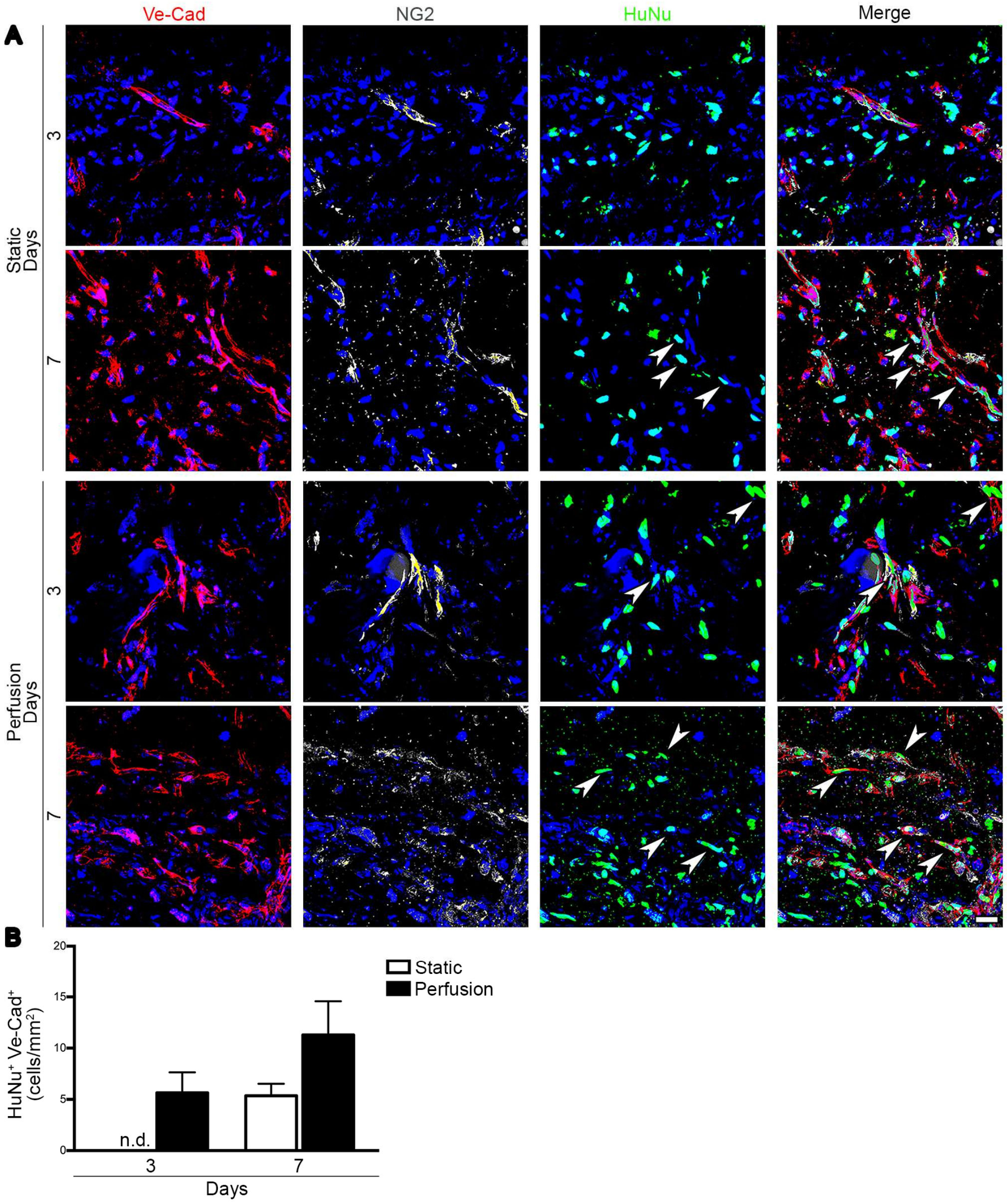

Supplementary Figure S6. At early time point human cells were not directly involved in the formation of blood vessels. (**A**) Representative immunofluorescence images for endothelial junctions (Ve-Cad; red), pericytes (NG2; gray) and HuNu (green). Nuclei were stained with DAPI (blue). White arrows indicate human cells Ve-Cad$^+$ or NG2$^+$. (**B**) Quantification of the human cells Ve-Cad$^+$ normalized over the analysed area. In static condition, at 3 days *in vivo*, the amout of HuNu$^+$ Ve-Cad$^+$ cells was not detectable (n.d.) (n donor = 3). Scale bar = 20 µm.

Supplementary Table S1 Human Angiogenesis Array coordinates

Factors							
**Pro-angiogenic**		**Anti-angiogenic**		**Pro-inflammatory**		**Reference/CTR**	
**Coordinat**	**Target**	**Coordinat**	**Target**	**Coordinat**	**Targe**	**Coordinat**	**Target/Contro**
A9, A10	Angiogenin	A5, A6	Activin A	C15, C16	IL-1β	A1, A2	Reference
A11, A12	Angiopoietin	A7, A8	ADAMTS-1	C17, C18	IL-8	F1, F2	Reference
A13, A14	Angiopoietin	A15, A16	Angiostatin/Plasminoge	C21, C22	Leptin	F23, F24	Negative CTR
A17, A18	Amphireguli	B13/B14	Endostatin/Collagen	C23, C24	MCP-		
A19, A20	Artemin	C13, C14	IGFBP-3	D1, D2	MIP-		
B1, B2	Coag.	D9, D10	Pentraxin-3 (PTX-3)				
B3, B4	CXCL16	D19, D20	Platelet Factor 4 (PF4)				
B5, B6	DPPIV	D23, D24	Prolactin				
B7, B8	EGF	E1, E2	Serpin B5				
B9, B10	EG-VEGF	E3, E4	Serpin E1				
B11, B12	Endoglin	E5, E6	Serpin F1				
B15, B16	Endothelin-1	E7, E8	TIMP-1				
B17, B18	FGF acidic	E9, E10	TIMP-4				
B19, B20	FGF basic	E11, E12	Thrombospondin-1				
B21, B22	FGF-4	E13, E14	Thrombospondin-2				
B23, B24	FGF-7	E17, E18	Vasohibin				
C1, C2	GDNF						
C3, C4	GM-CSF						
C5, C6	HB-EGF						
C7, C8	HGF						
C9, C10	IGFBP-1						
C11, C12	IGFBP-2						
C19, C20	LAP (TGF-						
D3, D4	MMP-8						
D5, D6	MMP-9						
D7, D8	NRG1- β1						
D11, D12	PD-ECGF						
D13, D14	PDGF-AA						
D16, D16	PDGF-						
D17, D18	Persephin						
D21, D22	PIGF						
E15, E16	uPA						
E19, E20	VEGF						
E21, E22	VEGF-C						
